# Conduct problems, hyperactivity, and screen time among community youth: Can mindfulness help?

**DOI:** 10.1192/j.eurpsy.2024.590

**Published:** 2024-08-27

**Authors:** S. Kim, S. Munten, N. J. Kolla, B. Konkoly-Thege

**Affiliations:** ^1^Waypoint Centre for Mental Healthcare, Penetanguishene; ^2^McMaster University, Hamilton; ^3^Centre for Addiction and Mental Health; ^4^University of Toronto, Toronto, Canada

## Abstract

**Introduction:**

While technology continues to evolve and the prevalence of screen-based activities is rising, limited studies have investigated the effect of various types of screen time on youth behavioural problems. Further, the influence of mindfulness intervention programs on behavioural problems beyond hyperactivity is largely understudied.

**Objectives:**

This study aims to address a research gap by examining the associations between four types of screen time and hyperactivity and conduct problems among community youth during the pandemic. The current study also aimed to investigate the efficacy of a mindfulness-based intervention in reducing hyperactivity and conduct problems.

**Methods:**

Community youth aged 12-25 from Ontario, Canada, were recruited between April 2021 and April 2022 (n=117, mean age=16.82, male=22%, non-White=21%). The Mindfulness Ambassador Program, a structured, 12-week, evidence-based intervention program, was offered live, online and led by two MAP-certified facilitators. We conducted linear regression analyses using pre-intervention data to examine the unique association between the four types of screen time and behavioural problems (hyperactivity and conduct problems). The efficacy of the MAP on adolescent hyperactivity and conduct problems was examined considering the three survey time points (pre-, post-, and follow-up) using a series of linear regression models utilizing the Generalized Least Squares (GLS) Maximum Likelihood (ML), unstructured model.

**Results:**

The average score for conduct problems was classified within the normal range, while the average score for hyperactivity was considered borderline at baseline. More than 5 hours of playing video games were significantly associated with increased conduct problems [β= -1.75, 95% CI=-0.20 – 3.30, p=0.03]. Accounting for age, sex, baseline mental health status, and screen time, the mindfulness intervention program significantly contributed to decreased hyperactivity at post-intervention compared to the baseline [β=-0.49, 95% CI=-0.91 to -0.08, p=0.02]. It was maintained at follow-up [β=-0.64, 95% CI=-1.26 to -0.03, p=0.04].

**Conclusions:**

Our findings suggest an adverse impact of excessive video gaming on behavioural problems among community youth and confirm that the trend remains the same. Considering the simplicity, brevity, non-invasive nature and other mental health benefits of the mindfulness intervention, we argue that the results are promising and worthy of further study and larger-scale implementation. Clinicians, parents, and educators should work collaboratively to provide developmentally appropriate strategies to moderate screen time spent on video games among youth.

**Disclosure of Interest:**

None Declared

